# Impaired Diastolic Function Predicts Improved Ischemic Myocardial Flow by Mechanical Left Ventricular Unloading in a Swine Model of Ischemic Heart Failure

**DOI:** 10.3389/fcvm.2021.795322

**Published:** 2022-01-12

**Authors:** Tomoki Sakata, Shin Watanabe, Renata Mazurek, Spyros Mavropoulos, Francisco Romeo, Kelly P. Yamada, Kiyotake Ishikawa

**Affiliations:** Icahn School of Medicine at Mount Sinai, Cardiovascular Research Institute, New York, NY, United States

**Keywords:** LV unloading, myocardial infarction, coronary blood flow, ischemia reperfusion injury, end-diastolic pressure-volume relationship

## Abstract

**Background:** Impact of mechanical left ventricular (LV) unloading on myocardial tissue perfusion and its regulating factors remain unclear. This study was conducted to elucidate the predictors of regional blood flow (RBF) improvement by mechanical LV unloading.

**Materials and Methods:** One to four weeks after percutaneous induction of myocardial infarction (MI), Yorkshire pigs (*n* = 15) underwent mechanical LV unloading using Impella CP. Hemodynamic parameters were collected prior to LV unloading. RBF in infarct, border and remote myocardium were measured by fluorescent microsphere injections before and 120 min after LV unloading.

**Results:** RBF showed variable responses to mechanical LV unloading. While infarct RBF improved in general (0.33 ± 0.13 to 0.42 ± 0.19 mL/min/g, *p* = 0.06), there were a few pigs that showed little improvement. Meanwhile, there were no clear trends in the border (1.07 ± 0.47 to 1.02 ± 0.65 mL/min/g, *p* = 0.73) and remote myocardial RBF (1.25 ± 0.52 to 1.23 ± 0.68 mL/min/g, *p* = 0.85). In the simple linear regression analysis, cardiac output, mean pulmonary arterial wedge pressure, mean left atrial pressure, minimum LV pressure, end-diastolic LV pressure, maximum dP/dt, slope of end-diastolic pressure-volume relationship (EDPVR) and end-diastolic wall stress were significantly associated with % change of infarct RBF. In the multiple regression model, slope of EDPVR and maximum dP/dt remained as independent predictors of infarct RBF change.

**Conclusion:** Steeper EDPVR and lower maximum dP/dt were associated with increased blood perfusion in the infarct area after LV unloading. Our data suggests mechanical LV unloading is more beneficial in post-MI patients with high diastolic pressure associated with increased LV stiffness and in those with worse cardiac contractility.

## Introduction

In the modern era where catheter-based therapies have become routine treatment, acute mortality for myocardial infarction (MI) has dramatically reduced. However, there have been an increasing number of patients who develop ischemic heart failure due to markedly reduced contractility after MI (1). Ischemic heart disease is a major risk factor for heart failure (2), and repeat hospitalization for heart failure not only progressively worsens cardiac function and reduces patients' quality of life, but also has a significant impact on the health care economy (3, 4). Therefore, there is an urgent need to establish new treatments that minimize myocardial damage in MI and prevent the development of subsequent ischemic heart failure.

The Impella (Abiomed Inc., Danvers, MA) is a percutaneously implantable left ventricular (LV) assist device that can assist cardiac output by pumping blood from the LV to the aorta via an axial flow pump. In addition to the beneficial effect on systemic circulatory support, it has been shown that active withdrawal of blood from the LV reduces the ventricular wall stress in failing hearts (5). In animal studies, the implementation of Impella in the acute phase of MI was shown to reduce myocardial infarct size (6–8). Reduced LV workload, which reduces myocardial oxygen demand, is considered to be the underlying mechanism of infarct size reduction with LV unloading. Meanwhile, the impact of mechanical LV unloading on oxygen supply, namely tissue blood flow, remains uncertain. Our previous study has shown that mechanical LV unloading using Impella reduces LV wall stress and increases regional blood flow (RBF) to the ischemic myocardium in the subacute phase of MI (9). However, the degree of RBF improvement was not uniform across animals and in the subsequent studies, we observed minimal improvement in the ischemic tissue RBF after mechanical LV unloading in some animals. In order to maximize the benefits of mechanical LV unloading for MI treatment, it is essential to understand what influences myocardial tissue perfusion and offers myocardial protection.

In this study, we hypothesized that mechanical LV unloading is more effective in certain patient populations and that hemodynamics prior to mechanical LV unloading can predict the change in RBF. The purpose of this study was to determine factors that predict RBF improvement in the ischemic heart before and after the implementation of mechanical LV unloading.

## Materials and Methods

### Study Design

All animal procedures were approved by the Institutional Animal and Use Committee at the Icahn School of Medicine at Mount Sinai, and the care of all animals complied with the Guide for the Care and Use of Laboratory Animals (National Institutes of Health publication No. 85–23, revised, 1996). The animals were acclimatized at the animal facility for at least 72 h before being enrolled in experiments.

A total of 15 Yorkshire pigs (seven male and eight female, 42.1 ± 3.5 kg) that survived percutaneous MI induction were included in the study. Pigs were recovered and mechanical LV unloading experiments were conducted at 1 week (*n* = 8), 2 weeks (*n* = 6), and 4 weeks (*n* = 1) after MI. For all procedures, pigs were intubated and ventilated with 100% oxygen under propofol (10 mg/kg per hour) anesthesia. After each mechanical LV unloading experiment, pigs were euthanized, the hearts were quickly excised, and the great vessels were dissected. The right and left atria (RA, LA) were carefully separated from the ventricular chambers. The right ventricular (RV) free wall was dissected from LV and each myocardial chamber was weighed separately. Intra-atrial and intraventricular septum were included in the LA and LV, respectively.

### Creation of Myocardial Infarction

The detailed protocol of percutaneous MI induction was described previously (9, 10). Intravascular access was established by percutaneous puncture of the femoral artery under ultrasound guidance. A 7-Fr guiding catheter was then advanced to the left coronary artery. After coronary angiography, a 0.014-inch coronary wire was advanced, and an over-the-wire coronary balloon (4.0 x 8 mm) was delivered to the proximal to mid left anterior descending artery (LAD). Transmural infarction was induced by occluding the coronary artery for 90–120 min followed by reperfusion.

### Mechanical LV Unloading

After echocardiographic and hemodynamic evaluations, Impella CP was inserted through separate percutaneously established access in the femoral artery. Anticoagulation was managed by intravenous injection of heparin. Full pump support was maintained for 120 min.

### Echocardiography

All pigs underwent two- and three-dimensional echocardiography before the initiation of mechanical LV unloading using Philips iE-33 ultrasound system (Philips Medical Systems, Andover, MA) (11). LV volumes and LV ejection fraction were measured in three-dimensional echocardiographic images using a semiautomated border detection software (Philips QLAB 3DQ Advanced, Philips Medical Systems). The two-dimensional echocardiographic studies included parasternal long-axis and short-axis views to measure LV and left atrial diameters and LV wall thickness. Two-dimensional four-chamber images were acquired from the subxiphoid window, and cross-sectional images of the LV were obtained from the right intercostal spaces. Peak early and late diastolic mitral flow velocity (E and A) were evaluated in accordance with American Society of Echocardiography/European Association of Cardiovascular Imaging recommendations (12).

### Hemodynamic Measurements

A Swan-Ganz catheter (Edwards Lifesciences, Irvine, CA) was advanced to the main pulmonary artery through femoral vein access. Cardiac output was measured in triplicate using the thermodilution method after confirmation of hemodynamic stability and during breath-hold. Left atrial pressure was obtained using the atrial septal puncture technique.

A high-fidelity pressure-volume loop catheter (Millar Instruments, Houston, TX) was inserted into the LV *via* the right carotid artery to collect LV pressure-volume measurements during steady state and transient inferior vena cava occlusion with short breath-holds. All pressure-volume data were analyzed using an iox2 application (Emka Technologies, Falls Church, VA). Alpha value and parallel conductance were calibrated using cardiac output derived from thermodilution method using a Swan–Ganz catheter and bolus injections of hypertonic saline.

### Myocardial Blood Flow Measurements

Regional myocardial blood flow was quantified using fluorescently colored microspheres as previously described (9). Briefly, 1 × 10^7^ polystyrene fluorescent microspheres (15 μm; Interactive Medical Technologies, Irvine, CA) were injected into the left atrium through trans-septal access via the femoral vein. Reference blood was withdrawn from a femoral artery sheath using a specialized pump (Harvard Apparatus, Holliston, MA) for 2 min at a rate of 2.9 mL/min. Microspheres with different wave lengths were used for measurements before and 2 h after LV unloading. The number of fluorescent microspheres trapped in the infarct (LV anterior wall), infarct border (lateral wall), and remote regions (posterior wall) were quantified by flow cytometric analysis. Regional blood flow (RBF) was calculated using the following formula:


$$\begin{array}{l} \text{RBF}\left(\text{mL}/\text{min}/\text{g}\right)=\left(\text{R}\times \text{lt}\right)/\left(\text{Ibr}\times \text{Wt}\right) \end{array}$$

where R is blood reference withdrawal rate (2.9 mL/min); lt and Ibr are fluorescent counts in the tissue and the blood reference sample, respectively; and Wt is the weight of the tissue sample (g).

### Statistical Analysis

Continuous variables are expressed as mean ± SD. A paired Student's *t*-test was used to compare the differences between before and after LV unloading. The Pearson's correlation test was used for all correlation analyses. To determine the independent contributing factors for infarct RBF change after mechanical LV unloading, a multivariable linear regression analysis was performed, adjusted by the significant factors in simple linear regression analyses. All analyses were conducted with JMP version 12.0 (SAS Institute Inc., Cary, NC), and *p* < 0.05 was the criterion for statistical significance.

## Results

### Regional Blood Flow Change Before and After LV Unloading

A total of 15 pigs successfully completed the LV unloading experiment. Echocardiographic and hemodynamic results before mechanical LV unloading are shown in Table 1.

**Table 1:** Echocardiographic and hemodynamic parameters before mechanical LV unloading.

	***n* = 15**
Male gender, *n* (%)	7 (47)
Body weight, kg	42.1 ± 3.5
**Echocardiographic data**
3D LV End-diastolic volume, ml	111.0 ± 14.2
3D LV End-systolic volume, ml	70.1 ± 15.5
3D Stroke volume, ml	40.9 ± 10.9
3D Ejection fraction, %	37.1 ± 10.1
LA diameter, mm	45.4 ± 4.6
E/A ratio	1.12 ± 0.35
**Hemodynamic data**
Cardiac output, l/min	3.7 ± 1.2
Stroke volume, ml	44.0 ± 13.5
Mean pulmonary arterial wedge pressure, mmHg	9.9 ± 5.1
Mean pulmonary arterial pressure, mmHg	20.7 ± 7.0
Mean left atrial pressure, mmHg	9.4 ± 5.5
Maximum LV pressure, mmHg	105.9 ± 22.5
Minimum LV pressure, mmHg	7.2 ± 3.8
End-diastolic LV pressure, mmHg	21.5 ± 7.6
End-systolic LV pressure, mmHg	96.2 ± 25.1
Maximum dP/dt, mmHg/sec	1,677 ± 611
Minimum dP/dt, mmHg/sec	−1,660 ± 549
tau 1/2, msec	22.7 ± 8.7
Stroke work, mmHg*ml	3,030 ± 1,515
Effective arterial elastance (Ea), mmHg/ml	2.58 ± 0.97
ESPVR, mmHg/ml	1.13 ± 0.75
EDPVR, mmHg/ml	0.60 ± 0.39
Preload recruitable stroke work, mmHg	35.7 ± 14.4
End-diastolic wall stress, kdynes/cm^2^	58.2 ± 23.1

*EDPVR, end-diastolic pressure-volume relationship; ESPVR, end-systolic pressure-volume relationship; LA, left atrium; LV, left ventricle*.

**Figure 1:**
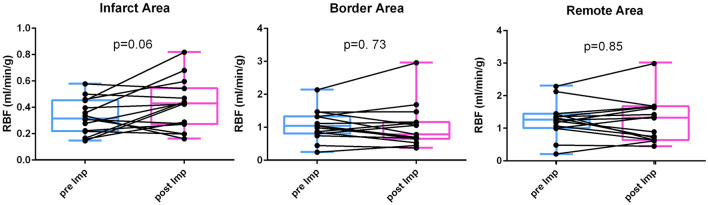
RBF change before and after LV unloading. Regional blood flow (RBF) was measured by fluorescent microsphere injection before and after mechanical LV unloading in the infarct, border and remote areas. After mechanical LV unloading, blood flow in the infarct area was increased whereas those in the border and remote areas did not show clear trends. LV, left ventricular.

Figure 1 shows the changes of RBF before and after mechanical LV unloading in infarct, border and remote myocardium at the papillary muscle level. While the majority of animals showed increased RBF in the infarct area, a few pigs exhibited little change. In contrast, no clear trends in RBF change were seen in other myocardial areas (Infarct RBF 0.33 ± 0.13 to 0.42 ± 0.19 mL/min/g, *p* = 0.06; Border RBF 1.06 ± 0.46 to 1.02 ± 0.65 mL/min/g, *p* = 0.73; Remote RBF 1.25 ± 0.52 to 1.23 ± 0.67 mL/min/g, *p* = 0.85).

### Linear Regression Analysis for Regional Blood Flow Change in Infarct Area

To determine the predictive factors associated with the RBF change in the infarct area, simple linear regression analysis was conducted on hemodynamic parameters before mechanical LV unloading. Table 2 shows the results of the regression analysis for % change of infarct area RBF. Cardiac output, mean pulmonary arterial wedge pressure, mean left atrial pressure, minimum LV pressure, end-diastolic LV pressure, maximum dP/dt and slope of the end-diastolic pressure volume relationship (EDPVR) were significantly associated with % RBF change.

**Table 2:** The results of the regression analysis for % change of infarct area RBF.

	**Standardized beta**	**Standard error**	**95% Confidential interval**	***p* value**
Cardiac output, l/min	−68.41	29.36	−131.82 – −4.99	0.04
Stroke volume, ml	−49.81	32.01	−118.95–19.33	0.14
Mean pulmonary arterial wedge pressure, mmHg	87.97	22.06	40.32–135.63	0.002
Mean pulmonary arterial pressure, mmHg	29.62	39.88	−56.53–115.77	0.47
Mean left atrial pressure, mmHg	78.49	23.57	27.57–129.41	0.005
Maximum LV pressure, mmHg	−46.19	32.87	−117.19–24.81	0.18
Minimum LV pressure, mmHg	91.47	24.82	37.83–145.11	0.003
End-diastolic LV pressure, mmHg	80.16	21.55	33.61–126.71	0.003
End-systolic LV pressure, mmHg	−42.28	34.81	−117.47–32.92	0.25
Maximum dP/dt, mmHg/sec	−65.45	26.24	−122.13 – −8.76	0.03
Minimum dP/dt, mmHg/sec	39.470	33.71	−33.37–112.30	0.26
tau 1/2, msec	−50.82	35.55	−127.62–25.97	0.18
Stroke work, mmHg*ml	−49.150	29.03	−111.87–13.58	0.11
Effective arterial elastance (Ea), mmHg/ml	20.37	40.79	−67.75–108.48	0.63
ESPVR, mmHg/ml	−30.58	32.26	−100.27–39.12	0.36
EDPVR, mmHg/ml	91.11	18.67	50.79–131.44	0.0003
Preload recruitable stroke work, mmHg	−48.67	27.50	−108.08–10.74	0.10

*EDPVR, end-diastolic pressure-volume relationship; ESPVR, end-systolic pressure-volume relationship; LV, left ventricle*.

To determine the independent predictive factors of % RBF change in the infarct area, multivariable linear regression analysis was conducted using the factors derived from simple linear regression analysis. Considering the collinearity, covariates which had strong correlation with other parameters (r > 0.8) were excluded and exchanged. To create the regression model, the forward stepwise method was applied on the following covariates: cardiac output, maximum dP/dt, mean pulmonary arterial wedge pressure, either of minimum or end-diastolic LV pressure, and slope of EDPVR. The model revealed maximum dP/dt and the slope of EDPVR before mechanical LV unloading as independent predictors of % RBF change in the infarct area. Both covariates were significant, and EDPVR was the strongest predictor (Table 3). These parameters remained significant when exchanging end-diastolic and minimum LV pressure.

**Table 3:** The result of multivariate linear regression analysis.

	**Standardized beta**	**Standard Error**	**95% Confidential Interval**	***p* value**
**R**^**2**^ **=** **0.776861, Adjust.R**^**2**^ **=** **0.739671**				
Maximum dP/dt, mmHg/sec	−43.14	16.32	−78.70 – −7.57	0.02
EDPVR, mmHg/ml	79.36	16.07	44.33–114.37	0.0003

*EDPVR, end-diastolic pressure-volume relationship*.

**Figure 2:**
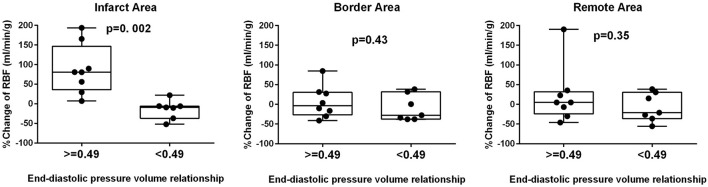
Diastolic dysfunction and RBF change. Comparison of percent change in regional blood flow (RBF) in the infarct, border and remote areas between high and low slopes of end-diastolic pressure-volume relationship. Significant difference was observed only in the infarct area.

The cutoff value of EDPVR slope for infarct RBF improvement, calculated from the ROC curve, was 0.49 (AUC 0.96, sensitivity 0.89, specificity 1.00). When pigs were divided into two groups according to the EDPVR slope prior to mechanical LV unloading (Figure 2), significantly higher % RBF change was found only in the infarct area of pigs with EDPVR slope greater than or equal to 0.49 (Infarct area *p* = 0.002; Border area *p* = 0.43, Remote area *p* = 0.35).

### Correlation Between Regional Blood Flow Change in Infarct Area and Tissue Weight

**Figure 3:**
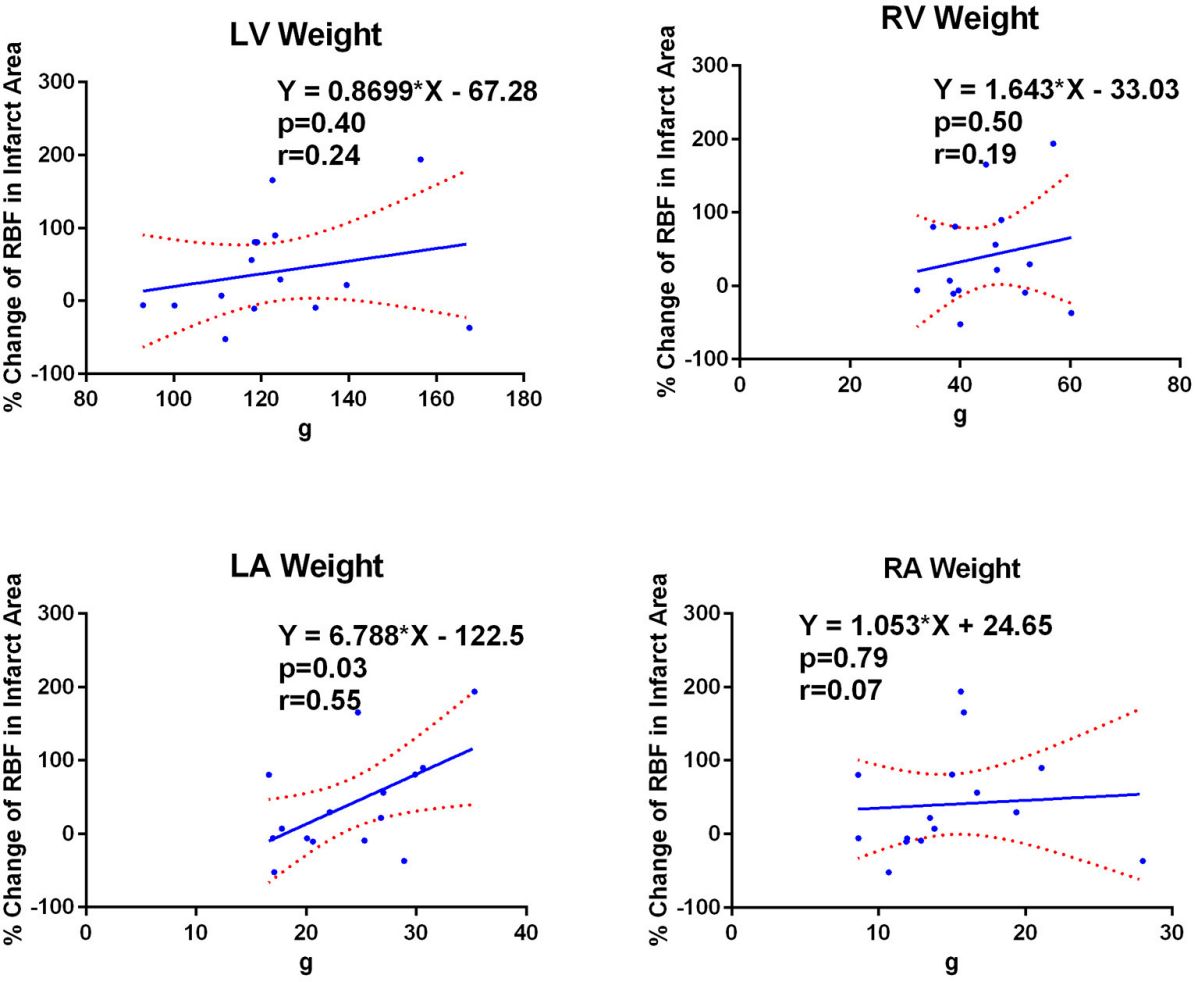
LA weight correlates with infarct RBF change. Correlations between percent regional blood flow (RBF) change in the infarct area and the weight of each cardiac chamber wall. Only the LA weight showed significant correlation to the percent RBF change in the infarct area. LA, left atrium; LV, left ventricle; RA, right atrium; RV, right ventricle.

Figure 3 shows the correlation between % RBF change in the infarct area and the weight of the RA, RV, LA and LV. Significant correlation was observed only with the LA weight (RA: 15.1 ± 4.8 g, r <0.01, *p* = 0.99; RV: 45.8 ± 8.0 g, r = 0.20, *p* = 0.45; LA: 23.1 ± 5.4 g, r = 0.58, *p* = 0.01; LV: 128.3 ± 20.4 g, r = 0.20, *p* = 0.44).

## Discussion

The main findings of the current study is that mechanical LV unloading improves myocardial tissue perfusion in the ischemic tissue and this improvement is most pronounced in animals with high EDPVR slope and low maximum dP/dt prior to unloading. High EDPVR slope indicates stiff heart whereas low maximum dP/dt is a sign of decreased cardiac contractility. These are commonly found in severe MI and our data suggest that mechanical LV unloading is more effective in improving ischemic tissue flow in those with severe heart failure.

Reduction of LV work has been highlighted as a mechanism of beneficial effect in mechanical LV unloading, whereas few studies examined how it influences tissue perfusion. We have previously shown that mechanical LV unloading improves ischemic myocardial tissue flow by reducing end-diastolic wall stress (9). This is an extension of our previous study with a focus on identifying the predictors of tissue perfusion improvement by mechanical LV unloading. Consistent with our previous data, mechanical LV unloading only improved perfusion in the infarct area in post-MI pigs. Several parameters that are associated with LV diastolic pressure and systolic function showed significant correlation to the percent RBF change in the infarct tissue. Multiple regression analysis demonstrated LV EDPVR slope and maximum dP/dt as independent predictors of infarct RBF change. Although both increased LV stiffness and impaired LV relaxation contribute to elevation of LV diastolic pressure, relaxation parameters such as tau 1/2 and minimum dP/dt were not significantly correlated to infarct RBF change. Meanwhile, end-diastolic parameters, such as end-diastolic pressure and EDPVR correlated with infarct RBF change, indicating the importance of diastolic distension in coronary flow regulation in the ischemic heart. By continuously withdrawing blood from the LV during diastole, Impella alleviates LV distension and increases infarct perfusion. The fact that only LA weight showed significant correlation to infarct RBF change while such correlation was absent in other chambers also supports the importance of LV distension, because increased LA weight is likely the result of continuously elevated LV diastolic pressure in these subacute MI animals.

In this study, not only diastolic distension but also impaired systolic contraction seemed to be a significant predictor of improved infarct RBF after Impella. This is likely associated with reduced cardiac output in those with low cardiac contractility, which can be augmented by Impella support. In fact, cardiac output was one of the predictive factors in univariate regression analysis although it's correlation to infarct RBF was less than that of maximum dP/dt. Whether reduced myocardial contractility *per se* contributes to low infarct RBF independent of reduced cardiac output needs further studies.

Even though LV pressure is equally applied to the infarct and non-infarct (remote) areas, the RBF change showed different patterns in each area. In the infarct area, higher slope of EDPVR and lower maximum dP/dt prior to mechanical LV unloading were associated with improved RBF, whereas RBF in the remote area did not show any clear relationship to these parameters. One possible explanation is the loss of auto-regulation in infarcted vessels due to endothelial dysfunction. Endothelial injury occurs in myocardial ischemia and subsequent ischemia-reperfusion injury (13–16). The damage is associated with neutrophil adhesion to vascular endothelial cells and reactive oxygen species production (17–19), which reduces the vasodilator reactivity (16, 20). Moreover, in the intact coronary circulation, the myogenic reaction plays an autoregulatory role in maintaining coronary blood flow, together with various vasodilatory agents released in response to metabolic demand (21–24). Without auto-regulation, the tissue perfusion likely becomes more dependent on extrinsic factors such as LV diastolic pressure and coronary perfusion pressure, which are modified by mechanical LV unloading using Impella.

### Clinical Implication and Future Perspectives

In acute and severe ischemic heart failure, inotropic therapy is often required for circulatory support. However, auto-regulation systems of coronary flow do not function effectively in the infarcted heart, and the mechanism of vasodilation that offsets the vasoconstriction of coronary microcirculation by inotropic agents does not work (17). This further worsens ischemia because blood supply to the myocardium is reduced despite higher myocardial demand associated with increased contraction. In such a situation, mechanical LV unloading can provide circulatory support while maintaining blood supply, which is a reasonable treatment for myocardial protection. Although our data was in subacute MI pigs, we expect that the same mechanisms are in place in acute MI and play key roles in myocardial protection that were described in previous studies (6, 25).

### Limitation

The microspheres technique allows sensitive quantitation of tissue perfusion, but lacks temporal information. LV pressure becomes the lowest just after aortic valve closing and rises gradually along with LV filling, thus the coronary flow is maximal at the early diastolic phase (26, 27). In our dataset, minimum LV pressure was also correlated to infarct RBF change, and whether Impella can lower minimum LV pressure and improve tissue perfusion also in the early diastole remains to be studied.

## Conclusion

Higher slope of EDPVR and lower maximum dP/dt before mechanical LV unloading predicted increased blood perfusion in the infarct myocardium. Our data suggests mechanical LV unloading is more beneficial in post-MI patients with high diastolic pressure due to increased LV stiffness and in those with worse cardiac contractility. Understanding the profiles of patients who would benefit more from mechanical LV unloading is essential for using this therapy in clinical ischemic coronary disease.

## Data Availability Statement

The original contributions presented in the study are included in the article/supplementary material, further inquiries can be directed to the corresponding author.

## Ethics Statement

The animal study was reviewed and approved by Institutional Animal and Use Committee at the Icahn School of Medicine at Mount Sinai.

## Author Contributions

TS and KI: conceived and designed research and interpreted results of experiments. TS: analyzed data, prepared figures, and drafted manuscript. KI: edited and revised manuscript. All authors performed experiments and approved final version of manuscript.

## Funding

This work was supported by National Institutes of Health R01 HL139963 (to KI), Japan Heart Foundation/Bayer Yakuhin Research Grant Abroad (to TS), and ABIOMED Inc. A-CURE Research Fellowship (to TS).

## Conflict of Interest

KI serves as a PI of a research grant to the institution from Abiomed Inc. TS received a research fellowship award from Abiomed Inc. RM received a research award from Abiomed. Inc. not related to this study. The remaining authors declare that the research was conducted in the absence of any commercial or financial relationships that could be construed as a potential conflict of interest.

## Publisher's Note

All claims expressed in this article are solely those of the authors and do not necessarily represent those of their affiliated organizations, or those of the publisher, the editors and the reviewers. Any product that may be evaluated in this article, or claim that may be made by its manufacturer, is not guaranteed or endorsed by the publisher.

## References

[1] Ezekowitz JA Kaul P Bakal JA Armstrong PW Welsh RC McAlister FA. Declining in-hospital mortality and increasing heart failure incidence in elderly patients with first myocardial infarction. J Am Coll Cardiol. (2009) 53:13–20. 10.1016/j.jacc.2008.08.067 19118718

[2] Benjamin EJ Virani SS Callaway CW Chamberlain AM Chang AR Cheng S. Heart disease and stroke statistics-−2018 update: a report from the american heart association. Circulation. (2018) 137: e67–492. 10.1161/CIR.0000000000000558 29386200

[3] Heidenreich PA Albert NM Allen LA Bluemke DA Butler J Fonarow GC. Forecasting the impact of heart failure in the United States. Circ Heart Fail. (2013) 6:606–19. 10.1161/HHF.0b013e318291329a 23616602 PMC3908895

[4] Lacey L Tabberer M. Economic burden of post-acute myocardial infarction heart failure in the United Kingdom. Eur J Heart Fail. (2005) 7:677–83. 10.1016/j.ejheart.2004.10.020 15921811

[5] Kapur NK Paruchuri V Urbano-Morales JA Mackey EE Daly GH Qiao X. Mechanically unloading the left ventricle before coronary reperfusion reduces left ventricular wall stress and myocardial infarct size. Circulation. (2013) 128:328–36. 10.1161/CIRCULATIONAHA.112.000029 23766351

[6] Kapur NK Qiao X Paruchuri V Morine KJ Syed W Dow S. Mechanical pre-conditioning with acute circulatory support before reperfusion limits infarct size in acute myocardial infarction. JACC Heart Fail. (2015) 3:873–82. 10.1016/j.jchf.2015.06.010 26541785

[7] Meyns B Stolinski J Leunens V Verbeken E Flameng W. Left ventricular support by catheter-mounted axial flow pump reduces infarct size. J Am Coll Cardiol. (2003) 41:1087–95. 10.1016/S0735-1097(03)00084-6 12679206

[8] Esposito ML Zhang Y Qiao X Reyelt L Paruchuri V Schnitzler G R. Left ventricular unloading before reperfusion promotes functional recovery after acute myocardial infarction. J Am Coll Cardiol. (2018) 72:501–14. 10.1016/j.jacc.2018.05.034 30049311 PMC6817809

[9] Watanabe S Fish K Kovacic JC Bikou O Leonardson L Nomoto K. Left ventricular unloading using an impella cp improves coronary flow and infarct zone perfusion in ischemic heart failure. J Am Heart Assoc. (2018) 7:e006462. 10.1161/JAHA.117.006462 29514806 PMC5907535

[10] Bikou O Watanabe S Hajjar RJ Ishikawa K. A pig model of myocardial infarction: catheter-based approaches. Methods Mol Biol. (2018) 1816:281–94. 10.1007/978-1-4939-8597-5_22 29987828

[11] Ishikawa K Aguero J Oh JG Hammoudi N Fish LA Leonardson L. Increased stiffness is the major early abnormality in a pig model of severe aortic stenosis and predisposes to congestive heart failure in the absence of systolic dysfunction. J Am Heart Assoc. (2015) 4:e001925. 10.1161/JAHA.115.001925 25994443 PMC4599422

[12] Nagueh SF Smiseth OA Appleton CP Byrd BF Dokainish H Edvardsen T. Recommendations for the evaluation of left ventricular diastolic function by echocardiography: an update from the american society of echocardiography and the european association of cardiovascular imaging. Eur Heart J Cardiovasc Imaging. (2016) 17:1321–60. 10.1093/ehjci/jew082 27422899

[13] Quillen JE Sellke FW Brooks LA Harrison DG. Ischemia-reperfusion impairs endothelium-dependent relaxation of coronary microvessels but does not affect large arteries. Circulation. (1990) 82:586–94. 10.1161/01.CIR.82.2.586 2372905

[14] Piana RN Shafique T Dai HB Sellke FW. Epicardial and endocardial coronary microvascular responses: effects of ischemia-reperfusion. J Cardiovasc Pharmacol. (1994) 23:539–46. 10.1097/00005344-199404000-00004 7516002

[15] DeFily DV Chilian WM. Preconditioning protects coronary arteriolar endothelium from ischemia-reperfusion injury. Am J Physiol. (1993) 265:H700–6. 10.1152/ajpheart.1993.265.2.H700 8368371

[16] Vanbenthuysen KM McMurtry IF Horwitz LD. Reperfusion after acute coronary occlusion in dogs impairs endothelium-dependent relaxation to acetylcholine and augments contractile reactivity in vitro. J Clin Invest. (1987) 79:265–74. 10.1172/JCI112793 3793926 PMC424037

[17] Muller-Delp JM. The coronary microcirculation in health and disease. ISRN Physiol. (2013) 2013:1–24. 10.1155/2013/238979

[18] Dreyer WJ Michael LH West MS Smith CW Rothlein R Rossen RD. Neutrophil accumulation in ischemic canine myocardium. Insights into time course, distribution, and mechanism of localization during early reperfusion. Circulation. (1991) 84:400–11. 10.1161/01.CIR.84.1.400 2060111

[19] Stewart DJ Pohl U Bassenge E. Free radicals inhibit endothelium-dependent dilation in the coronary resistance bed. Am J Physiol. (1988) 255:H765–9. 10.1152/ajpheart.1988.255.4.H765 3177668

[20] Kawachi Y Tomoike H Maruoka Y Kikuchi Y Araki H Ishii Y. Selective hypercontraction caused by ergonovine in the canine coronary artery under conditions of induced atherosclerosis. Circulation. (1984) 69:441–50. 10.1161/01.CIR.69.2.441 6690109

[21] Duncker DJ Koller A Merkus D Canty JM Jr. Regulation of coronary blood flow in health and ischemic heart disease. Prog Cardiovasc Dis. (2015) 57:409–22. 10.1016/j.pcad.2014.12.002 25475073 PMC5856234

[22] Pohl U Lamontagne D Bassenge E Busse R. Attenuation of coronary autoregulation in the isolated rabbit heart by endothelium derived nitric oxide. Cardiovasc Res. (1994) 28:414–9. 10.1093/cvr/28.3.414 8174163

[23] Kuo L Chilian WM Davis MJ. Interaction of pressure- and flow-induced responses in porcine coronary resistance vessels. Am J Physiol. (1991) 261:H1706–15. 10.1152/ajpheart.1991.261.6.H1706 1750529

[24] Kanatsuka H Lamping KG Eastham CL Dellsperger KC Marcus ML. Comparison of the effects of increased myocardial oxygen consumption and adenosine on the coronary microvascular resistance. Circ Res. (1989) 65:1296–305. 10.1161/01.RES.65.5.1296 2805245

[25] Ko B Drakos SG Ibrahim H Kang TS Thodou A Bonios M. Percutaneous mechanical unloading simultaneously with reperfusion induces increased myocardial salvage in experimental acute myocardial infarction. Circ Heart Fail. (2020) 13:e005893. 10.1161/CIRCHEARTFAILURE.119.005893 31959013

[26] Markwalder J Starling EH. A note on some factors which determine the blood-flow through the coronary circulation. J Physiol. (1913) 47:275–85. 10.1113/jphysiol.1913.sp001624 16993237 PMC1420483

[27] Hoffman JIE Buckberg GD. The myocardial oxygen supply:demand index revisited. J Am Heart Assoc. (2014) 3:e000285–e. 10.1161/JAHA.113.000285 24449802 PMC3959699

